# Metabolic Effects of FGF-21: Thermoregulation and Beyond

**DOI:** 10.3389/fendo.2015.00148

**Published:** 2015-09-25

**Authors:** Bin Ni, Jared S. Farrar, Janina A. Vaitkus, Francesco S. Celi

**Affiliations:** ^1^Division of Endocrinology and Metabolism, Department of Internal Medicine, Virginia Commonwealth University School of Medicine, Richmond, VA, USA

**Keywords:** FGF-21, fibroblast growth factor receptor, brown adipose tissue, metabolism, thermogenesis

## Abstract

Fibroblast growth factor (FGF)-21, a member of the FGF family, is a novel hormone involved in the control of metabolism by modulating glucose homeostasis, insulin sensitivity, ketogenesis, and promoting adipose tissue “browning.” Recent studies demonstrated that brown adipose tissue is not only a target for FGF-21, but is also a potentially important source of systemic FGF-21. These findings support the hypothesis that FGF-21 plays a physiologic role in thermogenesis and thermogenic recruitment of white adipose tissue by an autocrine–paracrine axis. This review examines the role of FGF-21 in thermogenesis from the perspective of cell-based, animal model, and human studies. We also present recent advances in the characterization of FGF-21’s regulation of metabolism.

## Introduction

Fibroblast growth factors (FGFs) are a large family of polypeptide growth factors comprised of 22 different members, with molecular masses ranging from 17 to 34 kDa sharing 13–71% amino acid identity. Among vertebrate species, FGFs are highly conserved in both gene structure and amino acid sequence (1). FGF-21 is a novel member of the FGF family and is predominantly expressed in the liver (2). FGF-21 activity depends on the membrane protein beta-klotho, a co-receptor with a very short cytoplasmic domain. FGF-21 binds beta-klotho with its C-terminus and directly interacts with FGF receptors (FGFRs) through its N-terminus, resulting in FGFR activation (3). During the past decade, FGF-21 has been shown to be a novel regulator of metabolism and a potential therapeutic target for the treatment of obesity and diabetes (4).

Adipose tissue is one of the main targets of FGF-21 action, and mammals have at least two different types of fat: white and brown. White adipose tissue (WAT) is the primary site of energy storage and of release of hormones and cytokines that modulate whole-body metabolism and insulin resistance (5, 6). Brown adipose tissue (BAT) is not only an important body defense against hypothermia but also plays a role in diet-induced thermogenesis (7). This energy expenditure is in the form of thermogenesis, and is mediated by the expression of uncoupling protein 1 (UCP1). Hence, BAT can regulate whole-body metabolism and may alter insulin sensitivity and modify susceptibility to weight gain (8, 9).

## FGF-21-Induced Thermogenesis in Mice

The first study of FGF-21’s function on mouse core body temperature was performed in 2005 by Kharitonenkov and colleagues (4). Transgenic mice expressing human *FGF-21* under the control of an *APOE* promoter and wild-type littermates were fed a high-fat/high-carbohydrate diet for 15 weeks. *FGF-21* transgenic mice were resistant to high-fat diet-induced obesity and their BAT was denser. At room temperature, *FGF-21* transgenic mice had significantly lower body weight, fasting glucose levels and leptin levels, despite a much higher food intake compared to wild-type littermates. These data suggest that in a fed state, FGF-21 probably stimulates EE partially through increased BAT activation, which in turn results in an improved metabolism.

A study on the role of the peroxisome proliferator-activated receptor alpha (PPAR-alpha)/FGF-21 endocrine signaling pathway found that FGF-21 could reduce physical activity and promote torpor, a short-term hibernation-like state of regulated hypothermia that conserves energy (10). This was due to direct *FGF-21* induction by PPAR-alpha in response to fasting, and pharmacologically by PPAR-alpha agonists. FGF-21, in turn, stimulated lipolysis in WAT and ketogenesis in the liver. In contrast to the previous study by Kharitonenkov and colleagues (4), the body temperatures of *FGF-21* transgenic mice in this study were 1–2°C lower than their wild-type littermates. To examine whether core body temperature was also affected under conditions in which endogenous FGF-21 was increased, wild-type mice were infected with an FGF-21 expressing adenovirus. Administration of FGF-21 resulted in reductions in both basal and fasting-induced body temperature. In addition to the reduced core body temperature, locomotor activity was also dramatically reduced in *FGF-21* transgenic mice. These data suggest that during fasting states, FGF-21 may alter behavior to conserve energy.

Coskun et al. investigated the role of FGF-21 in obese mice (11). Systemic administration of FGF-21 for 2 weeks in diet-induced obese mice and *ob*/*ob* mice lowered body weight by 20%, predominantly via a reduction in adiposity. In this study, FGF-21-treated animals exhibited increased EE, fat utilization, and lipid excretion, reduced hepatosteatosis, and ameliorated hyperglycemia. In contrast to previous studies, no decrease in total caloric intake or an effect on physical activity levels were observed. After continuous infusion with FGF-21 (1 mg/kg/day) for 5 days, a small but significant elevation of core body temperature was observed 4 h after initiation of treatment, lasting for approximately 10 h. FGF-21 also increased EE during both light and dark cycles, and depressed the respiratory quotient (RQ) in the dark cycles. The results of this study contrasts with an earlier report (10) where a reduction in body temperature was observed. It is important to note that in the former study, obese animals with *ad libitum* access to food were used and the body temperatures were measured in the fed state. Lower core body temperatures were only observed in lean, starved, and therefore energy-deprived *FGF-21* transgenic mice and no such effects were reported in fed animals. Collectively, the data indicate a divergent effect of FGF-21 on core temperature, driven by the fed/fasting state.

Subsequently, Hondares and colleagues investigated the thermogenic effect of FGF-21 on newborn mice (12). This manuscript for the first time provided evidence suggesting that a metabolic regulator hormone, produced by the liver, could directly mediate activation of brown fat thermogenesis during the fetal-to-neonatal transition. The authors found low levels of FGF-21 in the plasma of fetal mice. After birth, however, plasma FGF-21 levels increased rapidly and remained high for 6 days before declining. Once weaning had completed, FGF-21 levels were similar to adult mice. Similarly, *Fgf21* mRNA levels in both fetal and newborn mouse liver were low. Soon after birth, *Fgf21* was induced rapidly in the liver, with plasma FGF-21 levels reaching that of adults within 16 h. To assess whether activation of *PPAR-alpha* could mediate the action of milk lipids on the *Fgf21* gene, the authors studied *PPAR-alpha* null pups. Expression of *Fgf21* at birth in the liver of *PPAR-alpha* null mice was lower than their wild-type littermates. Moreover, postnatal induction of the *Fgf21* gene mediated by milk intake was completely suppressed in *PPAR-alpha* null pups. In wild-type pups starved after birth, injection of the PPAR-alpha agonist, pirinixic acid (Wy14,643) induced a significant increase in *Fgf21* expression levels in the liver, whereas no such response was achieved in *PPAR-alpha* null pups. These results, consistent with previous observations (10), indicate that PPAR-alpha is necessary for *Fgf21* induction in a fasting state.

Conversely, Hondares et al. (12) also found that FGF-21 could cause thermogenic activation of BAT in newborn mice and cultured brown adipocytes. The expression of thermogenic activation-related genes was increased in neonatal BAT after birth, in parallel with the rise in plasma FGF-21 levels. This study also treated neonate pups with a single dose of recombinant mouse FGF-21 4 h after birth, without further suckling. A small, but statistically significant reduction of blood glucose in the absence of differences in free fatty acids or β-hydroxybutyrate levels was observed. FGF-21 significantly induced the expression of genes encoding proteins involved in mitochondrial oxidation and BAT thermogenic activation, such as *Pgc-1*α*, Ucp1, Dio2, Cyt c, Lipe, Glut1*, *and Glut4*. Additionally, FGF-21 also increased the protein levels of peroxisome proliferator-activated receptor γ, coactivator 1α (PGC-1α), UCP1, and Cytochrome c. Moreover, *PPAR-alpha* null pups exhibited a reduced postnatal induction of thermogenic genes, which was rescued with FGF-21 treatment immediately after birth. Taken together, these results (12) confirm the role of *PPAR-alpha* in *Fgf-21* induction and FGF-21 activation of BAT thermogenesis in neonatal mice.

To assess whether FGF-21 may act directly on BAT, mouse brown adipocytes were differentiated in culture and were then exposed to FGF-21. At 5 nM, FGF-21 significantly induced the expression of genes involved in thermogenic activation (*Pgc-1*α*, Ucp1, Glut4, Cyt c*), while a higher concentration of FGF-21 (50 nM) did not further enhance these effects. Finally, mitochondrial function assays demonstrated that FGF-21 caused a significant increase in brown adipocyte oxygen consumption. FGF-21 treatment increased oligomycin-resistant uncoupled phosphorylation, and resulted in a significant induction (roughly threefold) of glucose oxidation in brown adipocytes. Additionally, these data indicated that FGF-21 specifically targets BAT thermogenic activation and that in neonates, this may contribute to neonatal activation of BAT thermogenesis in response to the start of milk intake. Collectively, these studies in mice demonstrate that FGF-21 is an important regulator of metabolism during fasting and fed states.

## Direct Release of FGF-21 by Brown Adipose Tissue

Although the liver is a well-known site for FGF-21 generation (2), Hondares et al. studied whether thermogenic activation in adult mice could directly induce FGF-21 expression and release in BAT (13). In this study, adult male mice were exposed to a 4°C housing environment for 6 or 24 h. This cold exposure caused a remarkable increase (40-fold) in *Fgf21* expression levels in BAT. Chronic acclimation to 4°C over 30 days also resulted in a significant induction of *Fgf21* expression. In contrast, *Fgf21* expression levels were not significantly increased in the livers of mice acutely exposed to cold and were even reduced in mice acclimated to cold during a 30-day exposure. In mice exposed to cold for 24 h, a modest increase in *Fgf21* mRNA was observed in WAT. Thus, whereas *Fgf21* mRNA levels in BAT at thermoneutrality were lower than in the liver and WAT, after thermogenic activation, BAT showed the highest levels of *Fgf21* mRNA expression. Plasma levels of FGF-21 in mice did not change after 6 h, but increased after 24 h of cold exposure, and increased even further after a 30-day cold acclimation period. The authors also found that there were significant negative arteriovenous gradients in FGF-21 concentration across interscapular BAT from rats after short term (24 h) cold exposure and long term (30 day) acclimation to cold exposure, demonstrating a significant FGF-21 output from BAT. Very recently, Keipert and colleagues showed that in UCP-1 knockout mice, cold exposure induces a dramatic increase in FGF-21 serum levels and FGF-21 expression in BAT compared to wild-type controls (14). This experimental model of ablation of non-shivering thermogenesis (NST) provided compelling evidence that FGF-21 acts in an autocrine–paracrine fashion on BAT, contributing to the adaptive thermogenic response and browning of WAT, ultimately expanding the NST capacity.

## Molecular Mechanisms of FGF-21-Induced Thermogenesis

The mechanisms which mediate the induction of *Fgf21* gene expression in response to cold were also investigated by Hondares et al. (13). Norepinephrine, a main mediator of cold-induced thermogenic activation of BAT, significantly induced *Fgf21* expression in cultured, differentiated mouse brown adipocytes. The same response was observed in brown adipocytes treated with cyclic adenosine monophosphate (cAMP). The induction of *Fgf21* gene expression by either norepinephrine or cAMP in brown adipocytes led to a significant increase in the release of FGF-21 into the cell culture medium. Treatment of brown adipocytes with propranolol, a β-adrenergic antagonist, successfully suppressed the effects of norepinephrine on *Fgf21* gene expression, whereas prazosin, an α-adrenergic inhibitor, had no effect. Furthermore, treatment with a protein kinase A (PKA) inhibitor (H89) or p38 mitogen-activated protein kinase (MAPK) inhibitor (SB202190) suppressed the induction of *Fgf21* gene expression by norepinephrine. These results indicate that norepinephrine acts through β-adrenergic receptors to increase cAMP levels, causing cAMP-mediated activation of PKA and p38 MAPK pathways and induction of *Fgf21* gene expression.

The authors also used HIB-1B cells [a brown adipocyte cell line derived from a transgenic mouse brown fat tumor (15)] to further investigate how *Fgf21* gene transcription was regulated. HIB-1B cells were co-transfected with a luciferase reporter construct with a *Fgf21* gene promoter and active form of PKA. By treating with dibutyryl cAMP, a significant increase in *Fgf21* promoter activity was found. Furthermore, both active (MKK6-Glu) and inactive (MKK6-K82A) forms of the upstream inducer of p38 MAPK, MKK6, were tested for their role in *Fgf21* promoter activity. Only MKK6-Glu could significantly induce *Fgf21* promoter activity after transfection in the cell, which indicated that PKA-mediated activation of *Fgf21* transcription is p38 MAPK dependent. Additionally, in a chromatin immunoprecipitation experiment, a very strong binding of ATF2 (cyclic AMP-dependent transcription factor) to the *Fgf21* promoter was observed, suggesting that cAMP can promote this binding event in brown adipocytes. These data provide a link between sympathetic activation during cold stress and the release of FGF-21 from brown adipocytes. Finally, this study also demonstrates that ATF2 binding to the *Fgf21* gene promoter is essential for cAMP-dependent induction of *Fgf21* gene transcription. Further studies investigating the binding of ATF2 to the *Fgf21* promoter may reveal novel regulators and the role of histone remodeling in *Fgf21* induction.

## FGF-21 Function in Human Brown Adipose Tissue

In 2013, our lab first discovered the effects of mild cold exposure on circulating FGF-21 levels in healthy humans and its relationship with cold-induced thermogenesis and lipolysis (16). Twelve healthy volunteers were included in a randomized, single-blind, crossover intervention study. All subjects were exposed to 24 and 19°C in a whole-room indirect calorimeter for 12 h. EE, plasma FGF-21, non-esterified fatty acid (NEFA), and adipose tissue glycerol concentrations were measured. First, we found that plasma FGF-21 exhibited a two- to threefold diurnal variation at 24°C, peaking at 0800 hours and progressively dropping to a nadir at 1700 hours, before rising at 1900 hours. We next compared in the same volunteers FGF-21 levels in blood samples obtained at 24°C and at 19°C. The diurnal rhythm of plasma FGF-21 concentration was retained at 19°C; however, the rise in FGF-21 levels in the late afternoon observed at 24°C was not. Plasma FGF-21 concentration was significantly higher at 19°C compared with 24°C at paired time points of cold exposure. Compared with 24°C, at 19°C the FGF-21 area-under-the-curve (AUC) was significantly increased. Also, the change in adipose tissue microdialysate glycerol concentrations between 19 and 24°C correlated positively with the change in plasma FGF-21 levels, but not the change in serum NEFA AUC. Total energy expenditure (TEE, or EE) was significantly higher at 19°C compared with 24°C, and the change in plasma FGF-21 concentration correlated positively with the change in EE. In a multivariate analysis, the change in plasma FGF-21 concentrations predicted the change in TEE. Collectively, our study demonstrated that mild cold exposure elevates circulating FGF-21 levels in humans. Augmented FGF-21 levels correlated positively with increments of adipose tissue microdialyzate glycerol and TEE during cold exposure. To our knowledge, this was the first study revealing potential regulatory links between FGF-21, lipolysis, and cold-induced thermogenesis in humans.

More recently, we explored cold exposure-induced FGF-21 secretion in humans and compared it with FNDC5 (also termed Irisin) (17). To elucidate whether BAT is a significant source of cold-augmented FGF-21 secretion, we profiled FGF-21 levels in five healthy men, stratified by BAT status, during 5 h of either a mildly cold non-shivering condition (19°C) or thermoneutrality (24°C). Consistent with our previous findings, diurnal reduction of FGF-21 was blunted at 19°C. However, the blunting effect was markedly greater in BAT-positive subjects compared to BAT-negative subjects, translating to a total FGF-21 output more than sixfold higher in BAT-positive individuals. Since the subjects were of similar age and body composition, and differed only by BAT status, these associative results support BAT as a source of FGF-21 during cold exposure in humans. Interestingly, the biorhythm of FGF-21 was virtually identical when subjects were exposed to warm temperature (27°C), and we observed a trend between FGF-21 diurnal reduction and shivering intensity (17).

We next examined the bioenergetic profiles of FGF-21-treated primary human adipocytes *in vitro*, which were established from deep neck fat biopsies, a location known to be enriched with beige adipocytes (18). FGF-21 treatment increased general BAT and beige gene expression, without altering expression levels of classic brown fat lineage genes. UCP1 protein, which was absent in untreated adipocytes, became strongly expressed following FGF-21 treatment. During an adipocyte thermogenic function assay, FGF-21 significantly enhanced adipocyte basal oxygen consumption rate measured with an extracellular fluid bioanalyzer. Pharmacological interrogation of mitochondrial respiration revealed augmentation of both forms of respiratory uncoupling (oligomycin resistant and maximal) by FGF-21 treatment. Since the primary function of BAT is the generation of heat, we quantified the heat production from adipocytes directly by infrared thermography. FGF-21 increased heat production only after norepinephrine exposure. Thus, not only norepinephrine is required for FGF-21 induction and release in BAT (13), it is also required for FGF-21-induced thermogenesis in BAT. In contrast, FNDC5 treatment enhanced adipocyte heat production dose dependently, and was further increased by norepinephrine (17).

Most recently, serum FGF-21 levels were correlated with BAT activity in humans (19). A total of 59 lean and healthy subjects were included in two different cooling protocols: air-cooling (20, 21) and water-perfused suit (22, 23), followed by a [^18^F]FDG-PET/CT scan as a measure for BAT activity. In agreement with our previous study, serum FGF-21 levels were positively correlated with human cold-induced BAT activity. Interestingly, this was observed only in men, although BAT is more likely to be detected in females than in males (24). In contrast to our previous studies, the authors did not find an effect of acute cold exposure on plasma FGF-21 levels, but did observe an increase after a 10-day cold acclimation period, in parallel with upregulation of BAT activity. These results further support a role for FGF-21 as a cold-induced activator and product of BAT. Additionally, basal FGF-21 levels correlated positively with the change in core body temperature upon cold exposure for both males and females in both the water-cooling group and the air-cooling group. This result suggests that in humans, FGF-21 may play a novel role in maintaining core body temperature in response to reduced ambient temperature.

## Metabolic Benefits of an FGF-21 Analog

In a clinical setting, elevated serum FGF-21 levels have been observed in obese, glucose intolerant, and diabetic patients (25–27). FGF-21 levels are positively correlated with adiposity, plasma glucose, and insulin levels (indices of insulin resistance and triglyceride (TG) levels), and negatively correlated with high-density lipoprotein cholesterol (HDL-C) (28). So far, no data are available on direct administration of native FGF-21 to humans; however, a FGF-21 analog (LY2405319) has shown promise in obese human subjects with type 2 diabetes (29, 30). LY2405319 is a biopharmaceutical optimization of native FGF-21 with enhanced physical stability, and is able to maintain biological potency *in vivo* more successfully than the native molecule (31). During a clinical study, LY2405319 (3, 10, 20 mg/kg/day) was injected subcutaneously for 28 days. Compared with placebo, the 10 and 20 mg groups showed reductions in low-density lipoprotein cholesterol (LDL-C) by 29.5 and 20.2%, respectively. An additional significant decrease in mean fasting TG levels appeared as early as day 2 for all three dosing groups. The reduction in fasting TG levels was maintained for the entire treatment period and was significantly different from baseline and placebo at the 10 and 20 mg dosage levels. Total cholesterol concentrations were also lowered in the 10 (−19.2%) and 20 mg/kg/day (−15.4%) groups. Additionally, an increase of 15–20% in HDL-C occurred across all three dosage groups. Compared with baseline, both 10 and 20 mg dosage groups had a significant decrease in body weight (−1.75 and −1.49 kg). There was a trend of lower glucose levels in all dosage groups, and fasting insulin levels were significantly reduced in the 20 mg dose group when compared to baseline. In mice, FGF-21 administration lowered body weight and increased metabolic rate without suppressing food intake (11). In the human clinical study, neither caloric intake nor EE were measured. However, the significant increase in β-hydroxybutyrate in all LY2405319-treated groups suggests that TEE might have been increased, since β-hydroxybutyrate has a substantial thermic effect in humans, leading to an increase in EE (32). Thus, it would appear that despite elevated baseline FGF-21 levels (“FGF-21 resistance”), obese individuals with type 2 diabetes may still benefit from administration of an FGF-21 analog. Future studies are still needed to determine the potential metabolic benefits of interventions, which increase endogenous FGF-21 levels in this population.

## Metabolic Benefits of FGF-21 may be “Browning” Independent

Brown adipose tissue has been increasingly recognized as a natural target for the modulation of EE. This tissue is very different from a white fat depot, in that it requires the uptake of free fatty acids and glucose for activation and expends energy instead of storing it (33). Although the most important functions of BAT in small mammals are thermogenesis and maintenance of core body temperature, more recent evidence has shown that this tissue may represent an important target in humans for the treatment of obesity (34–36) and type 2 diabetes (37–39). Due to FGF-21’s function as an important thermogenic regulator, its role in energy metabolism has also been widely investigated (4, 11, 40, 41). These *in vivo* studies used *ob/ob*, *db/db*, diet-induced obese, and *FGF-21* transgenic mice models. *FGF-21* transgenic mice had significantly lower body weight, fasting glucose levels, and hepatic fat deposition. They also retained more BAT, had subcutaneous adipocytes of smaller size, and exhibited improved glucose clearance and insulin sensitivity when compared to control littermates. Pharmacologic administration of FGF-21 dose dependently (0.1–10 mg/kg/day) reduced body weight and whole-body fat mass in diet-induced obese mice by increasing EE and physical activity levels. In diet-induced obese mice, FGF-21 also reduced blood glucose, insulin, and lipid levels, and reversed hepatic steatosis. Similarly, FGF-21 administration improved hepatic and peripheral insulin sensitivity in both lean and diet-induced obese mice, independent of a reduction in body weight and adiposity. Finally, it is worth noting that exogenous FGF-21 can promote reductions in fasting glucose levels, body weight, and circulating lipid levels in obese diabetic rhesus monkeys (42).

Recently, it has been debated whether the metabolic benefits of FGF-21 are “browning” and UCP1 dependent (43–45). These articles highlight a common pharmacological role for FGF-21 independent from BAT, since FGF-21 is effective in UCP1 null mice to reduce food intake for the promotion of weight loss. The majority of FGF-21-driven metabolic endpoints seem to not require UCP1 nor white adipocyte browning (brite or beige adipocytes); the browning of WAT by FGF-21 is dependent on temperature and diet, and UCP1 is only essential for helping FGF-21 regulate EE. These results are not surprising because FGF-21 acts through a cell-surface receptor complex composed of conventional FGFRs and beta-klotho (3, 46, 47). It is worth noting that both beta-klotho and FGFR1 are expressed not only in adipose tissue, but also in the liver and pancreas (48–50). Beta-klotho is also expressed in the suprachiasmatic nucleus (SCN) of the hypothalamus and the dorsal vagal complex (DVC) of the hindbrain in mice and this expression allows FGF-21 to regulate circadian behavior and metabolism at the level of the central nervous system (CNS) (51). Interestingly, the same study noted that the metabolic effects of FGF-21 overexpression to reduce blood glucose and insulin levels required beta-klotho expression in the SCN. This suggests that the CNS is pivotal in modulating the metabolic actions of FGF-21 in peripheral tissues, such as the liver and adipose tissue, and future studies are necessary to elucidate these pathways.

Skeletal muscle is also a target tissue for FGF-21 function (45, 52, 53). Skeletal muscle is strongly dependent on oxidative phosphorylation for energy production, and at the same time, skeletal muscle insulin resistance in type 2 diabetes and obesity involves dysregulation of the oxidation of both carbohydrate and lipid fuels. A recent study showed that in skeletal muscle from subjects with mitochondrial disorders, there was an increase in *FGF21* expression likely representing a compensatory mechanism (54). *FGF21* was induced in the skeletal muscle tissue of patients with mitochondrial oxidative phosphorylation deficiency and compensated for the metabolic energy deficiency by mammalian target of rapamycin (mTOR) activities, which serve as a master regulator of cell metabolism and energy homeostasis (55) via the PI3K–Akt pathway in skeletal muscle cells. mTOR can also increase the expression levels of *YY1* and *PGC-1*α, resulting in enhanced mitochondrial oxidative function. FGF-21-enhanced mitochondrial function was also demonstrated by significant increases in mitochondrial ATP synthesis, oxygen consumption rate, glycolytic capacity, activity of citrate synthase, and expression of key energy metabolism genes, such as *Glut1*, *Cpt1a*, *Cpt2*, *Cycs*, and *Idh3a*. All of these regulatory changes and recent insights into FGF-21 signaling in the brain contribute to the new role of FGF-21 in lipid and glucose metabolism, independent of thermogenesis or WAT “browning.”

## Concluding Remarks and Future Perspectives

**Figure 1:**
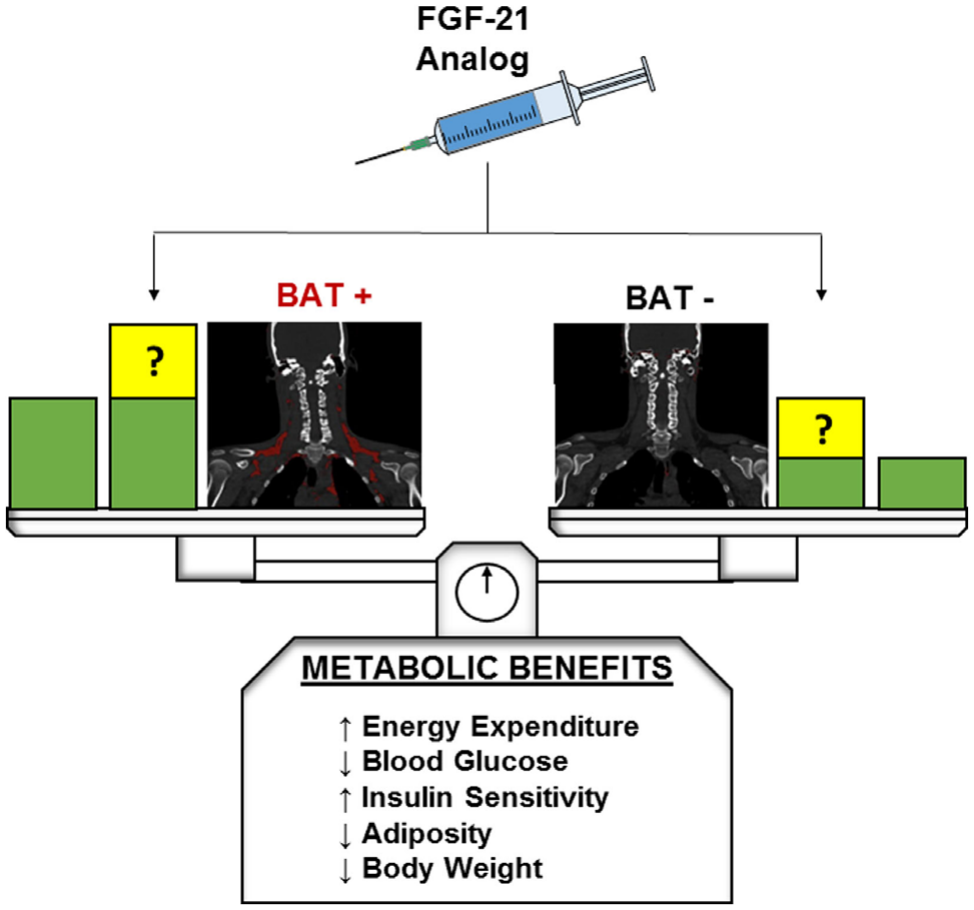
**Hypothetical metabolic benefits of FGF-21 in BAT-positive and BAT-negative individuals**. Green blocks indicate theoretical baseline metabolic benefits without FGF-21 or analog administration. At baseline BAT-positive individuals are expected to exhibit greater beneficial metabolic profiles than their BAT-negative counterparts. Yellow blocks indicate potential changes to baseline metabolic benefits after FGF-21 or analog administration. The presence and magnitude of these changes in both BAT-positive and BAT-negative patients have yet to be elucidated and are a target of future research.

Fibroblast growth factor-21 is a pleiotropic metabolic regulator which can reverse obesity and diabetes in different animal models and whose pharmacologic effects can be “browning” or UCP1-independent. In humans, a FGF-21 analog administration results in important metabolic effects, but further studies are required to elucidate the exact mechanisms of FGF-21 action. By taking advantage of dense phenotyping techniques with quantitative functional imaging of BAT, we hope to investigate whether or not individuals with greater initial BAT abundance or induced beige fat will have differential metabolic benefits during treatment with FGF-21 or its analogs (Figure 1).

## Conflict of Interest Statement

The authors declare that the research was conducted in the absence of any commercial or financial relationships that could be construed as a potential conflict of interest.
